# Case Report: Transformation From Cold to Hot Tumor in a Case of NSCLC Neoadjuvant Immunochemotherapy Pseudoprogression

**DOI:** 10.3389/fimmu.2021.633534

**Published:** 2021-02-17

**Authors:** Wenxiao Jia, Hui Zhu, Qianqian Gao, Jian Sun, Fujian Tan, Qun Liu, Hongbo Guo, Jinming Yu

**Affiliations:** ^1^Department of Radiation Oncology, Shandong Cancer Hospital and Institute Affiliated to Shandong University, Jinan, China; ^2^Department of Radiation Oncology, Shandong Cancer Hospital and Institute, Shandong First Medical University and Shandong Academy of Medical Sciences, Jinan, China; ^3^Department of Obstetrics and Gynecology, Qilu Hospital, Shandong University, Jinan, China; ^4^Department of Thoracic Surgery, Shandong Cancer Hospital and Institute, Shandong First Medical University and Shandong Academy of Medical Sciences, Jinan, China; ^5^BGI-Qingdao, Qingdao, China

**Keywords:** NSCLC, pseudoprogression, immunotherapy, imaging mass cytometry, single-cell RNA-sequencing

## Abstract

A 56-year-old male was diagnosed with right lung upper lobe squamous cancer with right hilar and mediastinum lymph node metastasis. After four cycles of neoadjuvant immunochemotherapy, reexamination by computed tomography showed progressive disease of the primary lesion. Then, the patient underwent a right lung upper lobectomy, and hilar and mediastinum lymph node dissection. Surgical pathology showed a partial response to immunochemotherapy. Single-cell RNA sequencing was used to characterize the infiltrating immune cell atlas after neoadjuvant immunochemotherapy; the most common infiltrating immune cell types were cytotoxic CD8+ T cells, monocyte-derived dendritic cells, and macrophages. Imaging mass cytometry revealed a transformation from cold to hot tumor after neoadjuvant immunochemotherapy. In this case study, we are the first to report a case of neoadjuvant immunochemotherapy pseudoprogression, proved by surgical pathology, single-cell RNA sequencing, and imaging mass cytometry. Both single-cell RNA sequencing and imaging mass cytometry revealed an activated immune microenvironment after neoadjuvant immunochemotherapy.

## Introduction

With the rapid development of immunotherapy, immune checkpoint inhibitors have become widely used for the treatment of metastatic and locally advanced non-small-cell lung cancer (NSCLC) (1–3). Owing to the activation of the patient's own immune cells to kill tumor cells (4, 5), several atypical response patterns, including hyperprogression, pseudoprogression, and dissociated response have been reported after immunotherapy, which are not observed in conventional cytotoxic antitumor treatment (6, 7). Pseudoprogression is a radiographic phenomenon in which an initial increase in tumor size is observed or new lesions appear, followed by a remission of the enlarged lesion; biopsy pathology of the enlarged lesion can be used to verify this phenomenon (8). In NSCLC patients treated with immunotherapy, the overall rate of pseudoprogression is 1.8–6.9% (6, 8). The exact mechanism of pseudoprogression is still not clear. In this study, we report a case of lung squamous cancer pseudoprogression after neoadjuvant immunochemotherapy. Pseudoprogression was proved by surgical pathology. Single-cell RNA sequencing (scRNA-seq) and imaging mass cytometry (IMC) were used to identify the exact infiltrating cell atlas after neoadjuvant immunochemotherapy, revealed a possible interaction between CD8+ T cells and CD14+ monocyte, CD16+ monocytes.

## Case Presentation

**Figure 1:**
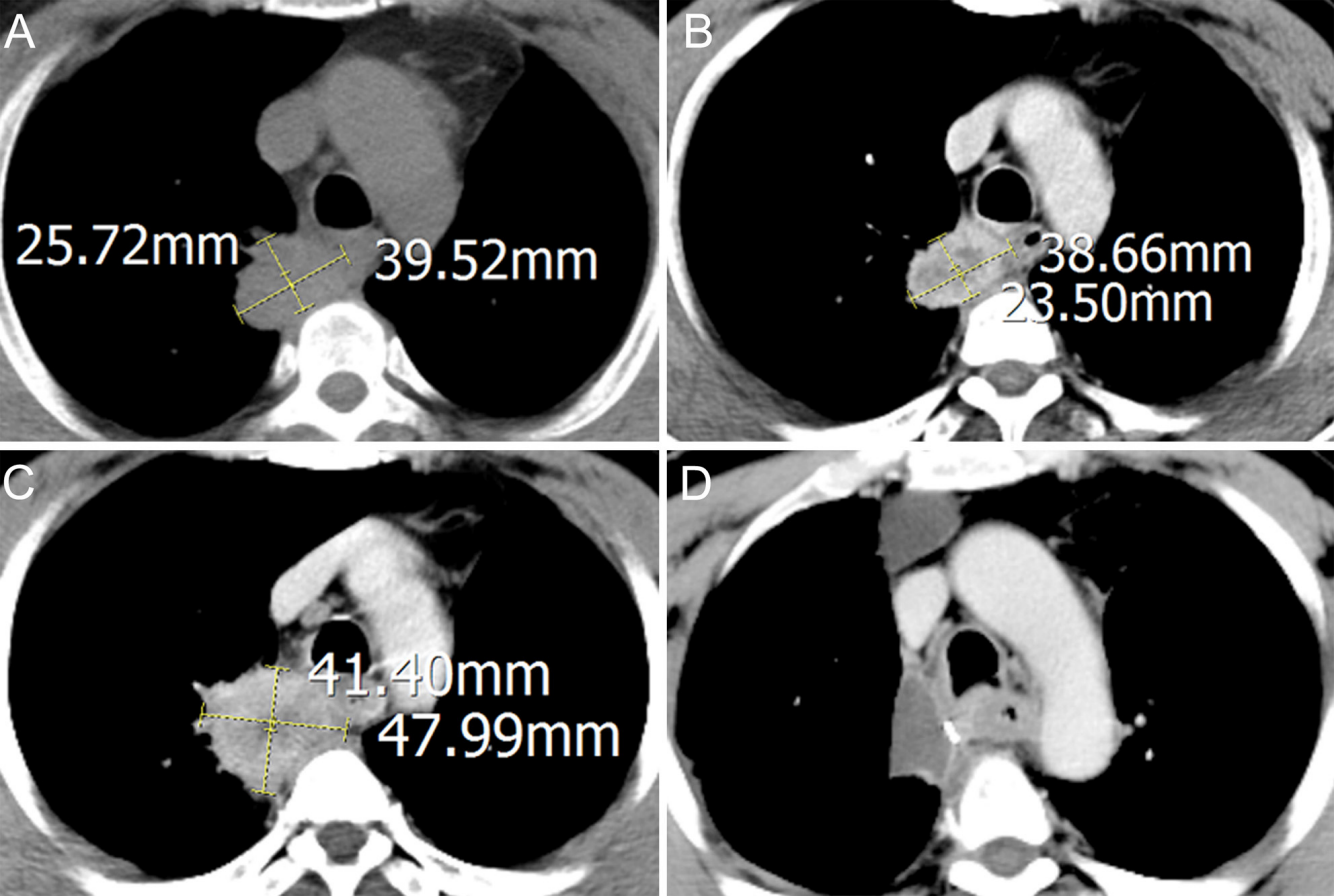
Computed tomography (CT) imaging of primary lesion at different times: **(A)** treatment-naïve; **(B)** after two cycles of neoadjuvant chemotherapy; **(C)** after another two cycles of neoadjuvant sintilimab combined with chemotherapy; **(D)** after operation.

A 56-year-old ex-smoker male was admitted to hospital for asymptomatic right lung mass and was diagnosed as right lung upper lobe squamous cancer with CT-guide lung biopsy, right hilar, and mediastinum lymph node metastasis was diagnosed with chest contrast enhanced computed tomography (CT) and the whole body positron emission tomography-computed tomography (PET-CT). The CT image of the primary lesion is presented in Figure 1A. After discussing in the multidisciplinary team (MDT), in view of the difficulty of operation, two cycles of neoadjuvant chemotherapy with gemcitabine and cisplatin were administered to the patient. Reexamination CT showed stable disease of the primary lesion after the two cycles of chemotherapy (Figure 1B). In order to reduce the difficulty of surgery operation further, another two cycles of neoadjuvant sintilimab (anti-PD-1 antibody) combined with gemcitabine and cisplatin were given to the patient, after which reexamination CT showed progressive disease (PD) of the primary lesion (Figure 1C), but the tumor biomarker, Cyfra21-1 continued to decline. After four cycles of neoadjuvant therapy, MDT discussion was organized again, and concurrent chemoradiotherapy was recommended in spite of the possibly of pseudoprogression, but the patient was rejected to radiotherapy and had strong willingness for operation. In view of the possibility of pseudoprogression, after a sufficient communication, the patient underwent a right lung upper lobectomy and hilar and mediastinum lymph node dissection; the postoperative CT image is presented in Figure 1D. Surgical pathology showed a partial response to immunochemotherapy, and the enlarged lymph nodes were presented as change after chemotherapy, no visible tumor cells existed. Now, the patient had finished all treatment and reexamination periodically for 13 months, no evidence of relapse or metastasis exist.

## scRNA-seq Reveals the Tumor Microenvironment After Neoadjuvant Immunochemotherapy

**Figure 2:**
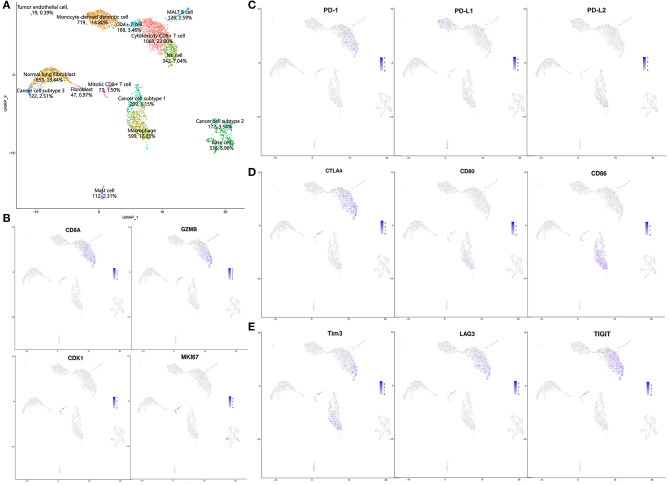
Results of single-cell sequencing: **(A)** UMAP results; **(B)** expression of CD8A, GZMB, CDK1, and MKI67; **(C–E)** expression patterns of immune checkpoints and corresponding ligands.

Given the possibility of pseudoprogression, we conducted scRNA-seq with the fresh surgical specimen. A total of 4,858 cells passed our quality filters. Unsupervised clustering analysis with Seurat identified 15 different cell clusters. The cell types in these clusters were annotated according to differentially expressed genes and the literature (9, 10). Based on the annotation results, we divided these cells into four categories: lymphocytes [cytotoxic CD8+ T cells: 22.00%, natural killer (NK) cells: 7.04%, CD4+ T cells: 3.46%, MALT B cells: 2.59%, mitotic CD8+ T cells: 1.50%]; myeloid-derived cells (monocyte-derived dendritic cells: 14.80%, macrophages: 12.33%, mast cells: 2.31%); stromal cells (normal lung fibroblasts: 13.44%, base cells: 6.96%, fibroblasts: 0.97%, tumor endothelial cells: 0.39%); and cancer cells (cancer cell subtype 1: 6.15%, cancer cell subtype 2: 3.54%, cancer cell subtype 3: 2.51%; Figure 2A). Complete pathological response (CPR) and major pathological response (MPR) can be used as surrogate markers to predict survival and are strongly associated with improved survival. Here, CPR was defined as no tumor cells existing after neoadjuvant therapy, and MPR was defined as <10% residual tumor (11, 12). In this patient, only 12.20% of cancer cells remained after neoadjuvant immunochemotherapy, nearly reached a major pathologic response, which was inconsistent with the radiological PD. Thus, we concluded that this might be a case of pseudoprogression.

The most common infiltrating cell types in this patient were cytotoxic CD8+ T cells and monocyte-derived dendritic cells, indicating an activated immune microenvironment after neoadjuvant immunochemotherapy (13, 14). We also identified a specific CD8+ T cell subtype with high expression of CD8A, GZMB, CDK1, and MKI67 (Figure 2B), indicating that this cell cluster consisted of active proliferative CD8+ T cells, which were annotated as mitotic CD8+ T cells (10). Expression of some immune checkpoint proteins were also observed, as shown in Figures 2C–E. In general, these results were consistent with those of previous reports: PD-1 and CTLA-4 were mainly expressed in CD8+ T cells and NK cells; regarding their ligands, PD-L1 was mainly expressed in monocyte-derived dendritic cells, whereas PD-L2 and CD80/86 were mainly expressed in macrophages. LAG3 and TIGIT were also mainly expressed in CD8+ T cells and NK cells, and Tim3 was mainly expressed in CD8+ T cells, NK cells, and macrophages (15, 16).

## IMC Reveals a Transformation From Cold to Hot Tumor After Neoadjuvant Immunochemotherapy

**Figure 3:**
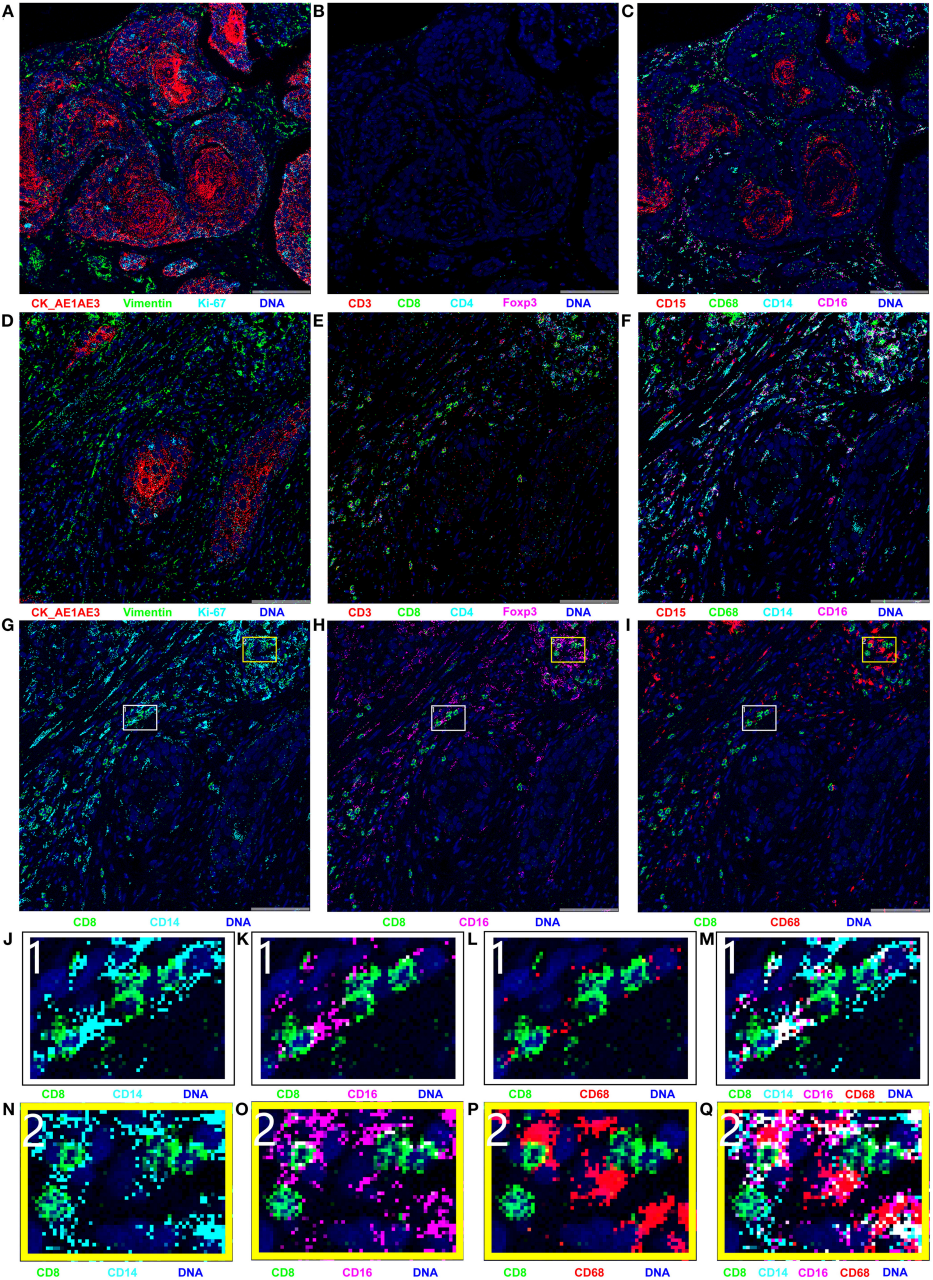
Results of imaging mass cytometry (IMC); all markers were stained in the same paraffin section and were present in different combinations. **(A–C)** IMC imaging of treatment-naïve specimen; **(D–Q)** IMC imaging of surgical specimen. The naïve specimen was mainly composed of cancer cells, with hardly any immune cell infiltration. Compared with the naïve specimen, the surgical specimen contained few cancer cells and was mainly infiltrated with CD8+ T cells, CD14+ monocytes, CD16+ monocytes, and CD68+ macrophages. **(G–I)** Spatial relationships between CD8+ T cells and CD14+ monocyte, CD16+ monocyte, and CD68+ macrophages. **(J–Q)** Enlarged versions of images in parts **(G–I)**.

IMC, an advanced technology that can be used to achieve simultaneous imaging of more than 30 proteins in a histologic section with a spatial resolution of 1 μm^2^, is a very useful tool in study of the tumor microenvironment and exploration of cell–cell interactions (17). In this study, IMC was performed on paraffin-embedded treatment-naïve and surgical specimens after neoadjuvant immunochemotherapy to explore the exact cell types infiltrating into tumor tissue. The IMC results are presented in Figure 3. In general, the IMC results in tumor tissue after neoadjuvant immunochemotherapy were consistent with the scRNA-seq results: only a few tumor cells existed, and the most common infiltrating immune cell types were CD14+ monocytes, CD16+ monocytes, CD8+ T cells, and CD68+ macrophages. Compared with treatment-naïve tumor tissue, the postoperative tumor tissue showed an obvious reduction in the number of tumor cells and expression of Ki-67 (Figures 3A,D). The most marked change was the increase in infiltration of lymphocytes, as shown in Figures 3B,E: in treatment-naïve tumor tissues, there were hardly any CD3+ T cells or CD8+ T cells, and only a few CD4+ T cells, whereas after neoadjuvant immunochemotherapy we found a large number of CD8+ T cells, the CD4+ T cells and CD3+ T cells are also increased in tumor tissue. Myeloid-derived cells also showed an obvious change (Figures 3C,F): compared with treatment-naïve tumor tissue, CD14+ monocytes, CD16+ monocytes, and CD68+ macrophages were markedly increased after neoadjuvant immunochemotherapy; in treatment-naïve tumor tissues, CD15+ neutrophils were mainly expressed in the tumor region, whereas after adjuvant immunochemotherapy, they were decreased and showed a diffuse distribution in the stromal region.

Besides the changes in tumor cells, T cells, and myeloid-derived cells, we observed a cell–cell contacts between CD8+ T cells and CD14+ monocytes, CD16+ monocytes in the tumor microenvironment after neoadjuvant immunochemotherapy. As shown in Figures 3G,H, CD8+ T cells were closely contacted with CD14+ monocytes, CD16+ monocytes in spatial position. The direct cell-cell contacts are shown more clearly in the enlarged images in Figures 3J,K,N,O, but no such phenomenon was observed between CD8+ T cells and CD68+ macrophages (Figures 3I,L,P).

## Discussion

Immunotherapy has shown great progress and become a new backbone of cancer therapy (1, 2). Pseudoprogression, an atypical response pattern, has gained increasing attention, but its exact mechanism has remained unclear (6, 8). In this study, we present a lung squamous cancer patient who was assessed to be “PD” on the basis of reexamination CT after four cycles of neoadjuvant immunochemotherapy; however, the surgical pathology indicated a partial response to immunochemotherapy, the scRNA-seq results showed that only 12.20% tumor cells remained, and IMC also showed few tumor cells remaining. Thus, we concluded that this was a case of pseudoprogression after neoadjuvant immunochemotherapy and showed that immune cell infiltration was responsible for the enlarged lesion.

In our previous review, we summarized some potential mechanisms of pseudoprogression and noted that hemorrhage, necrosis, edema, and infiltration of immune cells were the main reason for pseudoprogression (8), but until now, there still have little research focus on the exact molecular mechanism of pesudoprogression. The mechanism of pesudoprogression was mainly reported in case study, and the most commonly report was melanoma, NSCLC and renal cancer, the location of pseudoprogression was mainly reported in central nerve system and liver metastasis lesions (18–25). Summarize all of these case studies, we found the histopathology of pseudoprogression was mainly comprised of hemorrhage, necrosis, edema, and infiltration of immune cell, among them, the infiltrating immune cell attracted almost all attention. In 2018, Rocha et al. (21) reported a stage IV squamous cell lung cancer experience a pseudoprogression in liver lesions after five cycles of nivolumab, compared to the lung biopsy at diagnosis, the liver biopsy after pseudoprogression revealed an increased number of CD4, CD8, CD103 positive cells, the ratio of CD4/CD8 decreased to 0.875 from 1.25. In 2017, Curioni-Fontecedro et al. (22) reported a metastatic NSCLC treated with nivolumab, after six cycles nivolumab experience a metastatic lymph node pseudoprogression, and the biopsy revealed 60% CD3+ T cell and 30% CD19+ B cell infiltrate in tumor. Cohen et al. reported a patient experience melanoma brain metastasis pseudoprogression after pembrolizumab, the brain metastasis lesion was resected and the histopathology showed small clusters of tumor cells surrounded by hemorrhage and inflammatory cell, an abundance of CD68 positive microglial cells and CD45 positive cells were also scattered in inflammatory cell. In our study, scRNA-seq and IMC were first used to characterize the exact cell infiltration atlas after neoadjuvant immunochemotherapy pseudoprogression (18). According to the scRNA-seq results, the most common infiltrating immune cell types were cytotoxicity CD8+ T cells, monocyte-derived dendritic cells, and macrophages. We also identified a specific CD8+ T cell type, mitotic CD8+ T cells, with high expression of CDK1 and MKI67. IMC revealed a significant increase in infiltration of CD8+ T cells, CD14+ monocytes, CD16+ monocytes, and CD68+ macrophages after neoadjuvant immunochemotherapy, as well as obvious cell-cell contacts between CD8+ T cells and CD14+ monocytes, CD16+ monocytes in the tumor microenvironment. In general, the scRNA-seq results were consistent with the IMC results after neoadjuvant immunochemotherapy, with both indicating an activated tumor immune microenvironment.

According to both scRNA-seq and IMC, the most common infiltrating immune cell types after neoadjuvant immunochemotherapy were CD8+ T cells, monocytes, dendritic cells, and macrophages. The IMC results also revealed obvious cell-cell contacts between CD8+ T cells and CD14+ monocytes, CD16+ monocytes. As monocytes are the main antigen processing cell (26, 27), we speculated that CD14+ monocytes and CD16+ monocytes may present antigens to CD8+ T cells *in situ*. However, the antigen presentation process mainly occurs in secondary lymphoid organs; it has been unclear whether it also occurs in the tumor microenvironment (28). In this study, we provided evidence for *in situ* antigen presentation in the tumor microenvironment. The scRNA-seq results identified a specific T cell subtype, mitotic CD8+ T cells, which have previously only been reported in uveal melanoma; this subtype reflected the rapid proliferation status of CD8+ T cells and indicated that more cytotoxic CD8+ T cells were produced to execute cytotoxic functions (10). IMC revealed a transformation from cold to hot tumor after neoadjuvant immunochemotherapy in this patient (29). This phenomenon has also been observed in pancreatic cancer and NSCLC after neoadjuvant therapy (30, 31). The significantly increased infiltration of CD8+ T cells, CD14+ monocytes, CD16+ monocytes, and CD68+ macrophages represents an actived immune microenvironment. These results indicate that pseudoprogression patients can benefit from immunotherapy and provide a theoretical foundation for continuing immunotherapy in these patients.

In this study, we are the first time to report a neoadjuvant immunotherapy pseudoprogression case proved by surgical pathology, scRNA-seq, and IMC. Through scRNA-seq, we described the tumor-infiltrating immune cell atlas after neoadjuvant immunochemotherapy, and also identified a specific cell subtype, mitotic CD8+ T cells, which represent rapidly proliferative CD8+ T cells. IMC revealed a transformation from cold to hot tumor after neoadjuvant immunochemotherapy, with obvious cell-cell contacts between CD8+ T cells and CD14+ monocytes, CD16+ monocytes, providing evidence of *in situ* antigen presentation in the tumor microenvironment. In summary, this case study described the tumor infiltration immune cell atlas of a pseudoprogression patient after neoadjuvant immunochemotherapy, showed that the activated tumor immune microenvironment and provided a theoretical foundation for the continuation of immunotherapy in pseudoprogression patients.

## Data Availability Statement

The datasets presented in this study can be found in online repositories. The name of the repository and accession number can be found below: National Center for Biotechnology Information (NCBI) Sequence Read Archive (SRA), https://www.ncbi.nlm.nih.gov/sra/, SRP302765.

## Ethics Statement

The studies involving human participants were reviewed and approved by Shandong Cancer Hospital and Institute. The patients/participants provided their written informed consent to participate in this study. Written informed consent was obtained from the individual(s) for the publication of any potentially identifiable images or data included in this article.

## Author Contributions

All people who met authorship ICMJE criteria are listed as authors. HG and JY participated in the conception and design of this study. JS was involved in the operation. FT and QL performed the bioinformatic analysis. WJ, HZ, and QG collected and analyzed the data and drafted the manuscript. All authors contributed to the article, read, and approved the submitted version.

## Conflict of Interest

FT and QL were employed by BGI-Qingdao. The remaining authors declare that the research was conducted in the absence of any commercial or financial relationships that could be construed as a potential conflict of interest.
